# Promoting mental wellbeing in pregnant women living in Pakistan with the Safe Motherhood—Accessible Resilience Training (SM-ART) intervention: a randomized controlled trial

**DOI:** 10.1186/s12884-024-06629-2

**Published:** 2024-06-29

**Authors:** Shireen Shehzad Bhamani, An-Sofie Van Parys, David Arthur, Nicole Letourneau, Gail Wagnild, Olivier Degomme

**Affiliations:** 1 School of Nursing and Midwifery, Aga Khan University, Karachi, Pakistan; 2 Department of Public Health and Primary Care, Ghent University, Ghent, Belgium; 3 Bermi Acupuncture & Chinese Medicine Clinic, Bermagui, NSW Australia; 4 Peking Union Medical, Beijing, China; 5 Faculty OF Nursing, University of Calgary, Calgary, AB Canada; 6 Resilience Center, Montana, USA

**Keywords:** Resilience-building intervention, Perinatal mental health, Pregnancy, Pakistan

## Abstract

**Background:**

The negative impact of adverse perinatal mental health extends beyond the mother and child; therefore, it is essential to make an early intervention for the management of mental illness during pregnancy. Resilience-building interventions are demonstrated to reduce depression and anxiety among expectant mothers, yet research in this field is limited. This study aims to examine the effect of the ‘Safe Motherhood—Accessible Resilience Training (SM-ART)’ on resilience, marital adjustment, depression, and pregnancy-related anxiety in a sample of pregnant women in Karachi, Pakistan.

**Method:**

In this single-blinded block randomized controlled study, 200 pregnant women were recruited and randomly assigned to either an intervention or a control group using computer-generated randomization and opaque sealed envelopes. The intervention group received the SM-ART intervention consisting of six, weekly sessions ranging from 60 to 90 min. Outcomes (Resilience, depression, pregnancy-related anxiety and marital harmony) were assessed through validated instruments at baseline and after six weeks of both intervention and control groups.

**Results:**

The results revealed a significant increase in mean resilience scores (Difference:6.91, Effect size: 0.48, *p*-value < 0.05) and a decrease in depressive symptoms (Difference: -2.12, Effect size: 0.21, *p*-value < 0.05) in the intervention group compared to the control group. However, no significant change was observed in anxiety and marital adjustment scores.

**Conclusion:**

The SM-ART intervention has the potential to boost resilience scores and decrease depressive symptoms in pregnant women and offers a promising intervention to improve maternal psychological health.

**Trial registration:**

NCT04694261, Date of first trial registration: 05/01/2021.

**Supplementary Information:**

The online version contains supplementary material available at 10.1186/s12884-024-06629-2.

## Background

Pregnancy is a time of happiness, satisfaction, and pleasure for most women, while for some it is a source of stress, anxiety, and conflict [1, 2]. Meta-analyses of studies conducted on international samples of pregnant women reveal that one-fifth experience a mental health problem during their pregnancy [3, 4]. An umbrella review of 10 systematic reviews reported that antenatal depression ranges from 15 to 65%. Higher percentages are due to a higher burden in LMICs [5]. Another meta-analysis of 26 studies showed that 20.7% of pregnant women have an antenatal anxiety disorder [6]. Further, perinatal mental disorders are weighing on the health burden in lower and middle-income countries (LMIC) where estimates of mental illness are 20% and higher, predominating in the most vulnerable women—those deprived of accessibility to maternal and child health care [7, 8].

The prevalence of perinatal depression and anxiety in South Asia is among the highest in the world [9, 10], for example in Pakistan, the rates vary from 18 to 60% [11–14]. Women in these communities tend to hide their suffering due to stigmatization, shame, and fear of judgment by families and communities for seeking help from mental health services [15]. During pregnancy, intimate partner violence is also quite high [16]. According to a meta-analysis of 118 studies, the prevalence of any type of intimate partner violence during pregnancy was 25% worldwide and 32.1% in Asia [17]. Dennis et al. [18] in a meta-analysis of 21 studies, suggests that women who receive low income, lack social support, experience significant stress or negative life events, and have poor relationships are at higher risk of developing antenatal depression [18]. Pakistani women are more vulnerable to developing mental illness due to being overburdened by children and family responsibilities in extended families, as well as experiencing domestic violence and abuse emanating from cultural and societal patriarchal norms and values and also being disempowered or lacking decision-making power[19]. Supporting the hypothesis that populations in poorer countries and lower socioeconomic strata have higher incidences of mental health issues.

Depression and anxiety during pregnancy are also associated with a range of negative maternal and child health outcomes. These include pre-eclampsia, difficulties in performing daily activities, failure to seek prenatal care, inadequate diet and use of harmful substances (drugs, tobacco, and alcohol), postpartum depression, complicated birth, preterm birth, increased risk of fetal growth restriction and low birth weight [2, 3, 20–23]. The prevalence of these outcomes increases in low-income settings such as Pakistan [24].

Awareness of the increased risk of serious health outcomes during pregnancy is important, but effectively addressing and mitigating their impact requires more than just awareness. The antenatal period can cause an increased vulnerability to psychological distress, such as depression and anxiety, which can adversely affect both the mother and her unborn child [25]. Furthermore, women residing in LMICs face increased vulnerability to adverse outcomes as a result of distress, compounded by the limited availability and accessibility of mental health resources due to socioeconomic disadvantages [26]. Recognizing this, it has been acknowledged that fostering a positive outlook on life, effectively coping with emotions and challenges, and strengthening relationships can significantly contribute to mental well-being [27]. Timely intervention for addressing mental health issues during pregnancy is important; neglecting to address such issues may result in enduring consequences like postpartum depression and preterm birth or low birth weight. Rather than relying only on medical interventions, that may carry risks during pregnancy [28], it is important to prioritize interventions like resilience-building programs that enhance the positive outlook and coping skills in pregnant women. Resilience is the ability to navigate adversities, during pregnancy, this trait can enhance maternal well-being by enabling expectant mothers to cope with challenges [29]. It can reduce the negative impact of stress and depression, and maximize the wellbeing of a mother, her growing baby, and her family [30]. Promoting resilience during the antenatal period enables expectant mothers to develop adaptive coping mechanisms and emotional regulation skills, enhancing their ability to navigate the challenges of pregnancy and cultivate a more positive outlook, which is conducive to overall well-being. Thus, resilience-building interventions go beyond symptom management; they address the underlying factors contributing to psychological distress, such as social support, self-efficacy, and problem-solving skills. Moreover, resilience has the potential to mediate the impact of stress on psychological health by enhancing self-confidence to deal with adverse situations [31, 32]. Multiple meta-analyses have shown the effectiveness of positive psychological interventions in LMICs. These interventions, which include psychoeducation and emotional self-management delivered by trained health professionals, were found to be highly effective in improving mental health [33]. Similarly, psychosocial interventions during pregnancy, such as emotional self-management and social support, led to a decrease in common perinatal mental health disorders (depression, anxiety, and somatic issues) when implemented in community settings and antenatal healthcare facilities in low- and middle-income countries [35].

Additionally, the transition of pregnancy and the associated challenges can affect marital relationships. Unresolved mental health concerns during pregnancy can further strain and negatively impact marital relationships [36, 37]. However, building resilience can improve marital harmony by decreasing the detrimental effects of depression and enhancing the mental well-being of pregnant women [10]. Interventions aimed at enhancing resilience during this period not only empower women to communicate more effectively, resolving conflicts and expressing their needs and frustrations constructively, but also promote a deeper understanding of their own needs and encourage a proactive problem-solving approach to challenges, rather than resorting to blame. Building resilience is intertwined with marital harmony as resilient individuals are able to maintain healthy relationships even in the face of adversity. Moreover, marital harmony bears significance to safer parenting, as it contributes to a safer and nurturing family environment. Parents having stronger marital bonds through resilience-building interventions are better able to provide emotional support to their children [38].

Moreover, Bolier et al. [34] meta-analysis of 39 studies, reported that positive psychological interventions that build resilience, optimism, hopefulness, and wellness are effective in the enhancement of psychological wellbeing and managing situations that cause distress [34]. In a randomized controlled trial in Pakistan, Hirani, et.al (2017) reported that six weeks of social support intervention can significantly improve the resilience and quality of life of women (p < 0.05) [39]. Moreover, interventions that promote a positive approach and protective factors (optimism, resilience, mindfulness, social support) have been found to buffer the negative consequences of stress, anxiety, and depression, and maximize the wellbeing of a mother, her growing baby, and her family [30, 40]. The WHO strongly emphasizes the need to devote more attention to the prevention and promotion of mental health during pregnancy as these problems can result in lifelong health issues [41].

Considering all the interventions researched, resilience is one of the non-pharmacological approaches that benefits and helps an individual to acquire internal power, capacity, strengths, positivity, courage, competency, flexibility, and ability to cope effectively when faced with hardship [42]. Evidence suggests that resilience serves as a preventive factor against anxiety and depression during the perinatal period by mediating the impact of stress on psychological [31, 32].Thus, cultivating resilience can empower pregnant women with coping skills, enhancing mental well-being for themselves and their unborn children [4].

However, existing resilience frameworks often reflect Western cultural contexts [43–48], overlooking unique challenges faced in Pakistan, such as gender discrimination, male dominance, and deeply ingrained socio-cultural and religious beliefs. To address these needs, the SM-ART intervention was developed, drawing on contextually relevant attributes of resilience during pregnancy for women in Pakistan. It followed a systematic development process, incorporating insights from previous inquiries and content validation to ensure cultural relevance. This process guided the creation of a culturally and contextually relevant intervention aimed at promoting resilience among pregnant women [27, 49].

Since this hypothesis has yet to be tested in the Pakistani context, the study aimed to improve pregnant women’s resilience including her abilities to practice constructive coping, learn protective and proactive skills, and focus on positive adaptation for safe motherhood after participating in the SM-ART intervention (Safe Motherhood- Accessible Resilience Training). It is hypothesized that implementing the SM-ART intervention will not only reduce symptoms of depression and anxiety but also enhance individual resilience and marital harmony among pregnant women, thereby positively impacting the entire family dynamic.

## Methods

### Procedure

A single-center, single-blind, two-group Randomized Controlled Trial design (RCT) was adopted for hypothesis testing and to generate evidence in a rigorous and controlled condition to the degree possible [50]. The study was conducted at a midwifery-led clinic in Kohi Goth Hospital in Karachi, Pakistan.

Primary inclusion criteria were adult (18 +) pregnant women able to provide written consent and voluntary participation; a gestational age of between 12 and 30 weeks; and currently married and able to speak and understand the Urdu language. Participants were excluded if they had been diagnosed with any mental illness and/or physical illness to ensure that any observed effects can be attributed accurately to the intervention being studied. This was also done to prevent the outcomes of the intervention from being influenced by pre-existing mental or physical conditions, as well as the effects of medication taken for such conditions by expectant mothers [51, 52].

The sample size was calculated using NCSS PASS (2021) to detect non-inferiority using a one-sided, two-sample t-test. The margin of non-inferiority was -1.75 and a true mean difference of 3 between the resilience score of intervention and control groups with standard deviations of 10.300 and 9.900 respectively derived from a previous study[39]. The significance level (alpha) was set at 0.05 with a power of 80%. The total sample size was 114, with 57 participants allocated to each group. Anticipating potential attrition due to the study population and COVID-related challenges, the sample size was inflated by 40% and adjusted to 160 participants, with 80 participants allocated to each group. Moreover, to pilot the intervention, we sampled 20 more participants for each group, bringing the total sample size to 200, with 100 in each group.

Using permuted block randomization with blocks of four pregnant women, who were randomly assigned in a 1:1 ratio to either the intervention group (*n* = 100) or the control group (*n* = 100) to ensure equal representation of both groups in each block. After every five set of blocks, a sub-group consisting of 20 participants (10 in the intervention group and 10 in the control group) was formed. In total, 10 of these sub-groups were made, contributing to the overall sample size of 200 participants. The unit of randomization comprised individual pregnant women attending the clinic. The randomization list was generated using randomization computer software in the Clinical Trials Unit (CTU) of the Aga Khan University (AKU), Pakistan. The study arm allocation identities were sealed in opaque envelopes and kept by the research assistant. To eliminate selection bias, after obtaining informed consent and baseline data collection, the randomized envelopes were opened by the research assistant (MD) for the allocation to groups, which was then disclosed to the participants and further scheduling of post-assessment or intervention days were set accordingly. The randomization sequence list was not accessible to PI (SSB) and Co-PIs (DA, ASVP, NL, GW, OD). This encrypted file was retained at CTU until the end of the study. A rigorous oversight was upheld by the supervisory committee to maintain adherence to the protocol and prevent deviations. The CTU, functioning as an independent department, played an active role in overseeing the study, conducting thorough checks to ensure protocol compliance among all research staff.

**Fig. 1:**
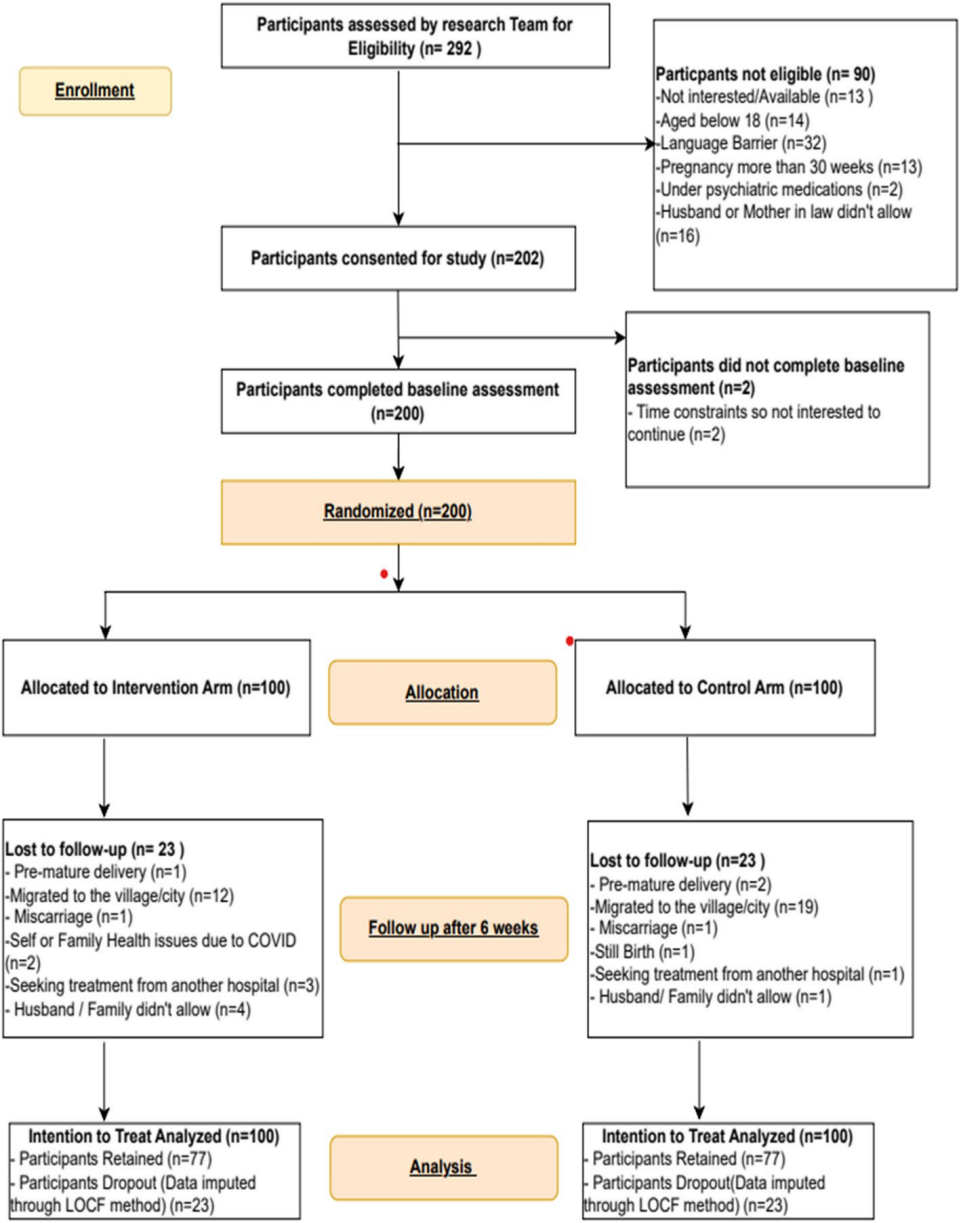
Study flow-chart

Potential participants were identified and recruited from the hospital clinic waiting areas by the CMWs, who assessed their eligibility. Upon determining eligibility, the CMWs referred eligible participants to the research team within the clinic. Eligible participants were provided with an explanation about the purpose, risks, benefits, and estimated time required for participation in the study. Those who agreed to follow the study procedures and provided written informed consent were enrolled (Fig. 1). Data collection for the intervention and control group were collected at two points: one at baseline and then after the intervention within two weeks of completion. The Principal Investigator (SSB) collected all baseline and post-intervention data while remaining blinded to the participants' group allocation. Data collection was conducted using a pencil-and-paper method within a private room at the clinic, ensuring privacy for each participant and ensuring identical conditions for both intervention and control group. Additionally, given the COVID-19 pandemic context at the time of the study, all standard operating procedures (SOPs) were strictly adhered to. This included maintaining a safe distance between the PI and the participant and wearing face masks throughout the interaction. All physical data were securely stored under lock and key, while electronic data were double-entered by independent personnel and password-protected for security.

Community Midwives (CMWs) from the clinics where the intervention was implemented were chosen to administer the intervention, aiming for a more feasible model. The midwives were experienced professionals who had undergone a one-year midwifery education training program following their completion of matriculation. They participated in a five-day training spread out over two weeks to accommodate their duties while ensuring comprehensive absorption and evaluation of the intervention content. The training, conducted by the PI, involved demonstrating and practicing specific teaching strategies related to the SM-ART intervention. Midwives were asked to demonstrate the complete training intervention within the group of trainees (CMWs) before delivering the actual intervention. This extensive training ensured thorough preparation, proficiency, and an in-depth understanding of the six intervention modules among the CMWs enabling them to confidently deliver the intervention. Additionally, the training covered the study's objectives, ethical considerations, privacy maintenance, confidentiality, and the importance of respecting participants and peers. The CMWs were additionally trained to recognize symptoms of depression, including sadness, crying, and feelings of hopelessness. They were equipped to identify these signs and intervene by involving the psychologist, who was a member of the team. Each session delivered by the midwives was supervised by a research assistant of the team unblinded to intervention allocation.

### Intervention group

Participants in the intervention group received a six-week group-based SM-ART intervention along with standard antenatal care by the clinic. The SM-ART intervention is a multifaceted, contextually and culturally appropriate intervention systematically developed through rigorous literature reviews and qualitative insights from key stakeholders, including pregnant women and mental health experts [27]. The research team, comprised of all study authors, including experts in mental health, maternal and perinatal health, thoroughly reviewed and developed this intervention content based on stakeholder input. This intervention was designed to promote resilience in pregnant women. The intervention was based on six theme-based training modules: Finding the Purpose of life, Dealing with Emotions, Believing in Yourself, Adapting an Optimistic Approach, Strengthening Support System and Relationships, Internalizing Spirituality and Humanity. These themes were identified in the formative phase of this study, as described in another article by PI [27, 49]. A comprehensive overview of the intervention's theoretical underpinning, development, validation process and included components are mentioned in a previous publication [49].

The intervention sessions included various teaching–learning strategies, including role plays, videos, group activities, brainstorming exercises, and scenario-based learning. Each session (six in total), lasting between 60 to 90 min, was conducted weekly, with one module delivered per week to groups of 10 participants.

To encourage participation, pick-up and drop-off transportation was offered. A takeaway lunch pack was provided after each session, and a babysitting facility was available with snacks and entertainment (such as coloring and storytelling books, balloons etc.) for the children. Regular reminders through phone calls and text messages for follow-up also encouraged compliance.

### Control group

Our control condition utilized "treatment as usual" (TAU) to assess the effectiveness of the intervention compared to standard current practices. Thus, the control group received the ‘standard antenatal care’ provided by the clinic during each of their antenatal appointments. These appointments included assessments such as blood pressure checks, weight monitoring, fetal growth tracking, ultrasound scans, and discussions about nutrition and exercise. This was consistent with the intervention group. However there was no formalized mental health care provided at Koohi Goth as part of any routine scheduled antenatal visits.

Following informed consent, control group participants underwent an initial baseline evaluation. Subsequently, participants received a reminder via phone call for the post-assessment, which occurred six weeks after the baseline assessment, aligning with the conditions of the intervention group's post-assessment.

Both intervention and control groups were given a mental health brochure and a complete list of references and local mental health services once after enrollment, which were encouraged to everyone for long term treatment. Weekly reminders through text messages and phone calls for follow-up (assessment or intervention session) also encouraged compliance.

### Measures

1. The Resilience Scale(RS-14) is a 14 items scale with a score range of 14–98 represents five characteristics of resilience based on the work of Gail since 1993 [53]: A purposeful life, Perseverance, Equanimity, Self-reliance, and Existential aloneness. Response choices are on a 7-point Likert scale ranging from 1 (Strongly Disagree) to 7 (Strongly Agree). A higher total score indicates higher resilience. The scale was validated for use in the Pakistani context by the PI [54].
2. The Edinburgh Postnatal Depression Scale (EPDS) is a 10-item; 4-point Likert scale with a score range of 0 to 30 to measure depressive symptoms. The EPDS was initially created as a postpartum measure of depression [55] and has now been validated for use in the perinatal period [56]. It has been used widely across 15 countries including some LMICs and validation studies of the tool have only recently been conducted in LMICs [57, 58]. Urdu scale validation demonstrates strong reliability and validity data (Cronbach’s α = 0.77; test–retest = 0.5) [59]. The higher total scores indicate more depressive symptoms.
3. Pregnancy-related anxiety (PRA) scale-revised is 10 items; a 4-point Likert-type scale with a score range of 0–3 that assesses anxiety associated with pregnancy [60]. The scales determine the mother's anxiety related to childbirth, fetal health, loss of fetus, confidence, own wellbeing, and parenting skills. The first five items are rated as not at all, somewhat, moderately, and very much. While items 6 through 10 are rated as never, sometimes, most of the time, and all the time, The scale was recently validated for use in the Pakistani context and showed acceptable reliability Cronbach’s α = 0.84, test retest = 0.45) [59]. There is no defined cutoff, so we consider scores as higher total scores indicate more anxiety.
4. The Locke-Wallace Marital Adjustment Test (15 Items) focuses on relationship aspects such as participation in shared activities, display of affection, and mutual spousal agreement on important matters related to marital adjustment. It is an extensively used self-report measure of adjustment in marriage. The scale scores range from 2–158. This scale is also validated in the Pakistani population [61]. Permission was sought from the researchers who have validated the tools.
5. Socio-Demographic data were collected with an instrument developed for this study, to measure variables that may affect the scores on depression, resilience, anxiety, and marital harmony. There were five sections: 1-Demographic factors (age, education, language, working status, etc.). 2-Pregancy related factors (gestational age, number of miscarriages, stillbirth, etc.), 3- Marriage related factors (duration of the marriage, choice of marriage, etc.), 4- Family-related factors (family type, husband employment, family income, etc.) and lastly variables related to her social life and management of emotional stress.

### Data analysis

With resilience as the primary outcome and marital satisfaction, pregnancy-related anxiety, and depression as secondary outcomes, data analysis was performed.

For descriptive statistics, data on key explanatory (predictor) variables on participants' demographic, pregnancy-related, marriage-related, and family-related, and social and economic characteristics were summarized by groups (Intervention versus Control). Chi-Square was used for categorical, while an independent t-test was used for continuous variables for between-group comparison. Fisher's Exact test was used for categorical variables where the expected frequency was < 5%, while the Mann–Whitney U tests were used if data were non-normally distributed. For reliability assessment, Cronbach’s alpha was used for all four scales at baseline (pre-intervention period) and six weeks after the intervention (post-intervention period) to measure internal consistency.

The analysis was conducted utilizing both Intention-to-Treat (ITT) analysis (Tables 6, 7, 8, 9, 10 and 11) and per-protocol analysis (Supplementary Table 5a-d) to comprehensively assess the efficacy of the intervention. To handle missing data for ITT analysis, the last observation carried forward (LOCF) method was utilized for data imputation where missing values were replaced with the previous most recent non-missing value observed for the same variable.

For inferential statistics, and as our primary analysis, the mean differences in primary and secondary outcomes were estimated between groups at baseline and post-intervention period, using independent t-test and paired t-test used to compare the mean difference between pre- and post-scores of all the outcomes among intervention and control groups. The effect size was also estimated for the post-intervention scores among the intervention and control group.

As a secondary analysis, we explored whether any demographic variable (individual or family-level) predicted the outcomes in the study sample using generalized linear modeling. Due to the nature of the study's experimental design, a difference score (Pre and Post) was adopted for each outcome variable as the dependent variable. Four linear regression models were built, one for each outcome. Several plausible interactions between independent variables and confounders were assessed. Effect estimates are reported in terms of beta coefficients and corresponding 95% confidence intervals and p values. Considering the one-sided hypothesis, the statistical significance was assessed using *alpha* 0.025. All the data were analyzed using SPSS v28.

## Result

Out of the 292 participants recruited, 90 were ineligible for study while two were not interested in continuing due to time constraints, thus a total of 200 participants were enrolled in the study. Among the 200 enrolled participants, 154 remained at post-assessment: 77 in both intervention and control groups—coincidently, the dropout rates in each group were similar (23%). Out of 100 participants enrolled in the intervention group, forty-five attended all six sessions of the intervention. Around thirty-three participants attended four to five sessions and eight individuals did not attend a single sessions after enrollment in the study. The average attendance in each sub-group ranges between 6.3 to 8.2 participants.

### Socio-demographic profile of study participants

There was no statistically significant difference in study participants' demographic, pregnancy-related, marriage-related and family-related, and social and economic characteristics except for the type of marriage. In the type of marriage, an increased frequency of arranged marriages (parent's choice determined spouse) in the control group was observed compared to the intervention group (95% versus 84%). Overall, the intervention and control group composition were balanced, which showed successful randomization (see Tables 1, 2, 3, 4 and 5). When comparing the basic demographic information from the dropouts to that of the remaining participants no significant difference was found. (refer Supplementary Table 1).

**Table 1:** Demographic profile of study participants

Demographic Factors	Intervention	Control	*p*-value
	*N* = 100	*N* = 100	
Age			0.32
< 20 years	18 (18.00%)	12 (12.00%)	
20–24	52 (52.00%)	47 (47.00%)	
25–29	19 (19.00%)	23 (23.00%)	
> 30	11 (11.00%)	18 (18.00%)	
Years of schooling			0.42
No formal education	30 (30.00%)	39 (39.00%)	
Primary (1–5 years)	27 (27.00%)	24 (24.00%)	
Secondary (6–10 years)	32 (32.00%)	31 (31.00%)	
Post-Secondary(> 10 years)	11 (11.00%)	6 (6.00%)	
Mother Tongue (a proxy for ethnicity)			0.70
Sindhi	11 (11.00%)	13 (13.00%)	
Urdu	26 (26.00%)	19 (19.00%)	
Punjabi	11 (11.00%)	10 (10.00%)	
Pushto	31 (31.00%)	32 (32.00%)	
Others	21(21.00%)	26 (26.00%)	
Financially Empowered			0.80
Yes	9 (9.00%)	7 (7.00%)	
No	91 (91.00%)	93 (93.00%)	

^*^signficant at < 0.005

**Table 2:** Pregnancy-related factors of study participants

*Pregnancy-Related Variables*	Intervention	Control	*p* value
	*N* = 100	*N* = 100	
Gestational age (in weeks)			0.57
Second Trimester	51 (51.00%)	47 (47.00%)	
Third Trimester	49 (49.00%)	53 (53.00%)	
First pregnancy (PrimiGravida)			0.88
Yes	29 (29.00%)	28 (28.00%)	
No	71 (71.00%)	72 (72.00%)	
History of miscarriage			0.63
Yes	26 (26.00%)	29 (29.00%)	
No	74 (74.00%)	71 (71.00%)	
History of stillbirth			0.44
Yes	2 (2.00%)	5 (5.00%)	
No	98 (98.00%)	95 (95.00%)	
Medical complications in current pregnancy (self-reported)			0.67
Yes	51 (51.00%)	48 (48.00%)	
No	49 (49.00%)	52 (52.00%)	
Intended or planned pregnancy			0.74
Yes	74 (74.00%)	76 (76.00%)	
No	26 (26.00%)	24 (24.00%)	

^*^signficant at < 0.005

**Table 3:** Marriage-related factors of study participants

*Marriage related variables*	Intervention	Control	*p*-value
	*N* = 100	*N* = 100	
Participant's type of marriage			0.019*
Arranged	84 (84.00%)	95 (95.00%)	
Self-choice	16 (16.00%)	5 (5.00%)	
Consanguineous marriage			0.39
Yes	57 (57.00%)	63 (63.00%)	
No	43 (43.00%)	37 (37.00%)	
Duration of marriage in years			0.092
1–2 years	42 (42.00%)	31 (31.00%)	
3–5 years	28 (28.00%)	26 (26.00%)	
6–10 years	24 (24.00%)	27 (27.00%)	
more than 10 years	6 (6.00%)	16 (16.00%)	

^*^signficant at < 0.005

**Table 4:** Family-related factors of study participants

*Family-related variables*	Intervention	Control	*p*-value
	*N* = 100	*N* = 100	
Participants spouse employed			0.59
Yes	91 (91.00%)	94 (94.00%)	
No	9 (9.00%)	6 (6.00%)	
Participants family type			0.091
Joint	75 (75.00%)	64 (64.00%)	
Nuclear	25 (25.00%)	36 (36.00%)	
Number of members in a household			0.84
Two people	10 (10.00%)	7 (7.00%)	
Three to five people	27 (27.00%)	31 (31.00%)	
Six to nine people	30 (30.00%)	29 (29.00%)	
More than ten people	33 (33.00%)	33 (33.00%)	
Number of Alive Children			0.56
1–2 Children	45 (45.00%)	39 (39.00%)	
3–5 Children	12 (12.00%)	17 (17.00%)	
> 5 Children	4 (4.00%)	7 (7.00%)	
Not applicable	39 (39.00%)	37 (37.00%)	
Household monthly income (Median and IQR)	17,500 (14, 000)	20,000 [110,000]	0.60
Own house			0.67
Yes	48 (48.00%)	45 (45.00%)	
No	52 (52.00%)	55 (55.00%)	
Own a vehicle for transportation			0.66
Yes	35 (35.00%)	38 (38.00%)	
No	65 (65.00%)	62 (62.00%)	

^*^signficant at < 0.005

**Table 5:** Social life and emotional management of study participants

***Social life variables***	**Intervention**	**Control**	***p*-value**
	*N* = 100	*N* = 100	
Number of friends (Mean & SD)	0.82 (1.32)	0.82 (1.75)	1.00
Number of friends			0.13
Zero	65 (65.00%)	75 (75.00%)	
1–2	23 (23.00%)	12 (12.00%)	
3 or more	12 (12.00%)	13 (13.00%)	
Participate in social or voluntary activities or services			0.54
No	96 (96.00%)	93 (93.00%)	
Yes	4 (4.00%)	7 (7.00%)	
***Emotional stress***			
Ability to manage financial demands			0.67
Yes	39 (39.00%)	42 (42.00%)	
No	61 (61.00%)	58 (58.00%)	
Feeling strain in marital life			0.77
Yes	7 (7.00%)	5 (5.00%)	
No	93 (93.00%)	95 (95.00%)	
Feeling strain in social life or with a family member			0.28
Yes	5 (5.00%)	10 (10.00%)	
No	95 (95.00%)	90 (90.00%)	

^*^signficant at < 0.005

### Reliability statistics of primary and secondary outcome measures

Cronbach’s alpha for all four scales ranged from 0.65 to 0.85 at baseline. They remained stable and similar over time, i.e., six weeks after the intervention. Resilience Scale-14 (Baseline: 0.73 and Post Intervention 0.80), Locke-Wallace Marital Adjustment Test (Baseline: 0.65 and Post Intervention 0.66), Pregnancy Related Anxiety Scale (Baseline: 0.71 and Post Intervention 0.73) and Edinburg Postnatal Depression Scale (Baseline: 0.85 and Post Intervention 0.85). This showed that all scales have acceptable to high internal consistent reliability.

### Comparison between intervention and control group at pre- and post-intervention level

Table 6 shows differences between intervention and control groups during pre-intervention and post-intervention periods. There was no statistically significant difference in the mean scores of the primary outcome resilience and secondary outcomes (marital satisfaction and pregnancy-related anxiety and depression) at the pre-intervention level. At post-intervention, mean resilience scores in the intervention group (82.68 ± 14.54) showed a significant difference (6.91) compared to the control group (75.77 ± 13.96), *P* < 0.05. Also the mean depression scores of the intervention group were (8.86 ± 6.67) significantly different (-2.12) compared to the control group mean (10.98 ± 7.56), *P* < 0.05. Thus, the results show that intervention group displayed significantly higher resilience (difference = 6.91, effect size = 0.48) and decreased depressive symptoms (difference = 2.12, effect size = 0.21) as compared to the control groups. However, there were no significant differences in the mean scores for marital adjustment and pregnancy-related anxiety between the intervention and control groups at the post-intervention level (see Table 6). Similar results were observed, with slightly higher estimates when drop-outs were excluded from the analysis (refer to Supplementary Table 2). Hence, our primary analysis reveals a significant change in resilience and depression following the SMART intervention, considering the pre- and post-assessment differences between intervention and control groups.

**Table 6:** Changes in primary and secondary outcome scores after receiving the SMART intervention and comparison by pre and post

	Pre-Intervention			Post-Intervention		
	**Intervention**	**Control**	***p-value***	**Intervention**	**Control**	***p-value***
	*N* = 100	*N* = 100		*N* = 100	*N* = 100	
***Outcomes***	Mean (SD)	Mean (SD)		Mean (SD)	Mean (SD)	
Resilience (14–98)	75.25 (13.54)	74.23 (14.34)	0.606	82.68 (14.54)	75.77 (13.96)	0.001*
Marital Adjustment (2–158)	121.00 (29.18)	125.67 (22.02)	0.203	123.02 (28.47)	123.57 (24.76)	0.884
Pregnancy-Related Anxiety (0–30)	15.23 (6.33)	15.85 (6.42)	0.493	13.79 (6.04)	14.39 (6.32)	0.494
Depression (0–30)	11.48 (7.53)	11.99 (7.38)	0.629	8.86 (6.67)	10.98 (7.56)	0.037*

Ϯ Independent t-test

^*^signficant at < 0.05

### Comparison within intervention and control group at pre- and post-intervention level

There was a statistically significant difference in the mean scores of resilience, depression, and pregnancy-related anxiety in the pretest compared to their respective post-mean scores in the intervention group. However, no significant difference was observed in the post-marital adjustment scores from their pre-scores in the intervention group. In the control group, there were no significant differences found in resilience, marital adjustment, and depression scores but there was a significant difference in the mean pregnancy-related anxiety score between pre-intervention to post-intervention time (Table 7). Hence our analysis reveals a significant change in the post-assessment scores of pregnancy-related anxiety in both intervention and control groups whereas significant changes in the post-intervention scores of resilience and depression were only found in the intervention group.

**Table 7:** Changes in primary and secondary outcome scores within each group after 6 weeks

	Intervention			Control		
	Pre	Post	*p value*^Ϯ^	Pre	Post	*p value*^Ϯ^
	*N* = 100	*N* = 100		*N* = 100	*N* = 100	
***Outcomes***	Mean (SD)	Mean (SD)		Mean (SD)	Mean (SD)	
Resilience (14–98)	75.25 (13.54)	82.68 (14.54)	< 0.001*	74.23 (14.34)	75.77 (13.96)	0.215
Marital Adjustment (2–158)	121.00 (29.18)	123.02 (28.47)	0.284	125.67 (22.02)	123.57 (24.76)	0.308
Pregnancy-Related Anxiety (0–30)	15.23 (6.33)	13.79 (6.04)	0.01*	15.85 (6.42)	14.39 (6.32)	0.010*
Depression (0–30)	11.48 (7.53)	8.86 (6.67)	< 0.001*	11.99 (7.38)	10.98 (7.56)	0.075

Ϯ paired t-test

^*^signficant at < 0.05

### Generalized linear regression

Four different multilinear regression models emerged from the regression modelling aiming to identify protective and risk factors for each outcome.

#### Model 1 (difference in resilience score)

After adjusting for baseline resilience scores, the increase in resilience score was on average 6.28 points more in the intervention group than the control group (p < 0.001). In addition, our findings show that a higher resilience score at baseline, the lower the increase in resilience at follow-up (β = -0.38; P < 0.001). (Table 8) Finally, we also tested interactions between group variables and resilience at baseline, but it remained not significant.

**Table 8:** Variables significantly predicting Resilience Score difference (n = 200)

			*95% CI*		
*Variables*	*Beta Coeff*	*SE*	*LL*	*UL*	*Pvalue*
Intercept	29.81	4.42	21.15	38.47	0.00*
Assigned Group					
Intervention	6.28	1.60	3.15	9.41	0.00*
Control (Ref)	0.00				
Resilience Score at Baseline	-0.38	0.06	-0.49	-0.27	0.00*

^*^signficant at < 0.05

#### Model 2 (difference in depression scores)

After adjusting for baseline resilience and depression scores, the decrease in depression score was on average 1.73 points more in the intervention group than in the control group (*p* < 0.05). In addition, our findings suggest that higher the resilience score at baseline, the lower were the depression scores at follow-up (β = -0.079 unit; *P* < 0.05). Moreover, the higher the depression scores at baseline, the lower the depression score at follow-up (β = -0.394 unit; *P* < 0.001) (Table 9). Finally, we also tested for interactions between group variables and depression at baseline, but it remained insignificant.

**Table 9:** Variables significantly predicting Depression Score Difference (n = 200)

			*95% CI*		
*Variables*	*Beta Coeff*	*SE*	*LL*	*UL*	*P-value*
Intercept	9.590	2.322	5.039	14.141	0.000
Assigned Group					
Intervention	-1.730	0.725	-3.152	-0.308	0.017
Control (Ref)	0.000				
Resilience Score at Baseline	-0.079	0.027	-0.132	-0.024	0.004
Depression Score at Baseline	-0.394	0.051	-0.494	-0.294	0.000

^*^signficant at < 0.05

#### Model 3 (difference in marital adjustment scores)

After adjusting for baseline marital adjustment and resilience scores, problems in current pregnancy and working status, the increase in marital adjustment score was on average 2.68 points more in the intervention group than the control group (*p* < 0.05). In addition, our findings suggest that the higher the resilience scores were at baseline, the higher marital adjustment scores were at follow-up (β = 0.30 unit; *P* < 0.001). However, the higher the marital adjustment scores were at baseline, the lower the marital adjustment scores were at follow-up (β = -0.33 unit; *P* < 0.001) (Table 10). In addition, the decrease in the marital adjustment score was on average 7.33 points more in those who had problem in their current pregnancy compared to those who did not (*p*-value = 0.033) and the decrease in marital adjustment score was on average 10.65 points more for those who were working compared to non-working women (*p*-value = 0.023).

**Table 10:** Variables significantly predicting Marital Adjustment Score Difference (n = 200)

			*95% CI*		
*Variables*	*Beta Coeff*	*SE*	*LL*	*UL*	*P-value*
Intercept	21.79	8.07	5.97	37.60	0.007
Assigned Group					
Intervention	2.68	2.48	-2.17	7.53	0.279
Control (Ref)	0.00				
Resilience Score at Baseline	0.30	0.10	0.12	0.49	0.001
Marital Adjustment Score at Baseline	-0.33	0.05	-0.44	-0.23	0.000
Working Status					
Yes	-10.65	4.68	-19.82	-1.48	0.023
No (Ref)	0.00				
Problems in current pregnancy					
Yes	-7.33	2.48	-12.19	-2.48	0.033
No (Ref)	0.00				

^*^signficant at < 0.05

#### Model 4 (difference in anxiety scores)

Table 11 depicts the modelling outcome for the difference in anxiety scores. After adjusting for baseline anxiety, depression score, problems in current pregnancy and the current pregnancy decision, the decrease in anxiety score was on average 0.30 points more in the intervention group than the control group (*p* < 0.05). In addition, our findings suggest that the higher the anxiety score at baseline, the lower in anxiety scores at follow-up (β = -0.47 unit; *P* < 0.001). However, the higher the depression score at baseline, the higher the anxiety scores at follow-up( β = 0.12 unit; *P* < 0.001) Moreover, the increase in anxiety score was on average 1.93 points more in those who had problems in their current pregnancy compared to those who did not (*p*-value < 0.001), and the decrease in anxiety score was on average 1.62 points more if the decision of being pregnant was the woman’s own choice compared to those where the husband made the decision (*p*-value = 0.03).

**Table 11:** Variables significantly predicting Anxiety Score Difference (n = 200)

			*95% CI*		
*Variables*	*Beta Coeff*	*SE*	*LL*	*UL*	*Pvalue*
Intercept	4.85	1.11	2.67	7.02	0.000
Assigned Group					
Intervention	-0.30	0.65	-1.57	0.97	0.64
Control (Ref)	0				
Anxiety Score at Baseline	-0.47	0.06	-0.59	-0.35	0.00
Depression Score at Baseline	0.12	0.05	0.02	0.23	0.02
Problems in current pregnancy					
Yes	1.93	0.66	0.63	3.23	0.00
No (Ref)	0				
Pregnancy Decision					
Own	-1.62	0.76	-3.11	-0.13	0.03
Husband (Ref)	0				

^*^signficant at < 0.05

## Discussion

In this study, we investigated whether women who participated in a six-week SM-ART intervention scored higher on resilience, and marital adjustment and reported lower depression and anxiety scores as compared to those who were in the control group. According to a recent report, Pakistan's maternal and child healthcare services are not up to standard with international norms [62]. Given the current evidence indicating potential fetal harm and epigenetic changes across generations due to maternal stress such as increasing fetal stress sensitivity, and, shorter gestational ages, particularly in LMICs where mental health if often neglected., there is an urgent need to prioritize mental health promotion within the Maternal and Child Health (MCH), which is currently a neglected part of the “Safe Motherhood”. Initiatives like SM-ART can address this issue, showing the potential for positive advancements in mental health promotion within MCH [63–65].

Primarily, the SM-ART intervention showed a positive increase in resilience scores among our study participants, aligning with previous research on positive psychosocial interventions. These interventions, including practices such as mindfulness and social support, have consistently shown to reduce distress among women and provide robust support for those in vulnerable circumstances [39, 66].

Secondly, our intervention showed a statistically significant reduction in depressive symptoms. A recent study conducted in China also supports this finding, where resilience was identified as a protective factor against depressive symptoms during pregnancy [67]. The higher resilience and lower depression scores in our intervention group can be explained by certain hypotheses for example, our group-based intervention gave the participants access to engage in peer support groups where they could practice active listening, express their emotions in a safe setting, and build trusting relationships with other participants. This process of connecting is supported by a study demonstrating that social interaction during pregnancy empowered women, enhanced interpersonal relationships, and helped develop effective coping methods for dealing with stressful situations and ultimately decrease stress, anxiety, and depression [68–70].

Thirdly, no significant differences were found in marital adjustment scores across the intervention and control groups. In context of our subcontinent, within Pakistan, evidence suggests societal acceptance of violence against married women and animosity in married life. Women are not raised to believe that being treated equally and respectfully is a fundamental right. Due to established pseudo-gender roles, blurred cultural and religious boundaries, and financial dependency on husbands, they are socialized to justify violence [71]. This might explain why we found similar scores in both groups given that there was no variation in the socio-cultural factors. Another explanation is that their male partners were not involved in the intervention, although the questionnaire did inquire about spouse responses. One of our six sessions did focus on improving the quality of their relationships at current stage as well as strategies to make it stronger, which may have influenced participants in a direct way but not necessarily their male partner.

Fourthly, our results showed that both intervention and control groups significantly reduced their post-intervention scores of pregnancy-related anxiety. Both groups were dealing with COVID 19 problems, and this could have increased their general level of anxiety linked with the uncertainty affecting their entire families and society. Literatures supports the notion that pregnant women are more vulnerable to specific stressors including fear of losing family and social support, changing delivery plans, fear of food running out, increased conflict in the home, fear of getting infected and loneliness, due to restricted activities of COVID-19 [72, 73]. Our study found inconsistent results, where pregnancy related anxiety scores decreased. This may be because our tool measures specific questions related to pregnancy related anxiety nor the general anxiety. Another aspect that could explain the decrease in fear is linked to our study-approach, where RA made a telephone call to the women in the intervention and control groups on a weekly basis to ask them certain questions about their general health. This was mainly done as a reminder of their upcoming intervention session or post assessment and considered to be an important ethical aspect during COVID time (second peak). Moreover, both the groups received a mental health brochure, with a list of referrals. This might also have an impact on the anxiety levels of the participants. Hence, we may conclude that these actions may reduce the anxiety score in both the groups.

Our predicting models showed that participant’s baseline resilience and depression scores were significantly associated with all outcomes including resilience, depressive symptoms, marital harmony, and pregnancy related anxiety. It follows that participants' baseline resilience and depression scores may have an impact on their post-intervention scores. Accordingly, if the SM-ART intervention is repeated and if the time between subsequent pregnancies is not too long, it may have an impact on future pregnancies as well. Previous studies in China showed that several factors during a first pregnancy might influence the improved outcome of a second pregnancy. For instance, mutual decision making between partners, improved financial condition, social support during delivery, family environment and perceived stress during the pregnancy [74]. Likewise, our SM-ART intervention may have a potential to be a sustainable intervention in Pakistan and throughout a woman’s pregnancy. In addition, statistical analysis of assessing interaction between baseline resilience or depression scores with the “group: intervention/control” variable revealed no significant association, so it means SM-ART is not only effective for ‘at risk’ groups but also with the ‘healthy’ general population. Furthermore, the small to medium effect size on depressive symptoms and resilience has the potential to improve a women's mental health. Hence, to address the mental health problems and its consequences during pregnancy with a resilience enhancing intervention like SM-ART should be promoted [75]. Especially in resource constrained countries such as Pakistan where there is a significant treatment gap, and access to mental health services is severely limited, an intervention such as SMART can be of added value. To minimize the burden of mental illness, it should be a top priority to adopt and promote prevention-based interventions that are affordable, non-pharmacological, individual-strength-focused, evidence-based, and accessible.

Additionally, it was also found that certain participants in each intervention group exhibited assertiveness, tenaciousness and stronger coping skills, which may have inadvertently intimidated other women who were more hesitant to share their difficulties. Studies suggest that participants in group sessions tend to develop hope, establish supportive social networks, draw inspiration from role models and overcome stigma [76, 77]. Our onsite psychologist and CMWs also noticed positive group dynamics throughout the intervention phase. One incident quoted by the psychologist involved participants engaging in discussions about their lives and routines beyond the scope of pregnancy and the research study. Additionally, she noted an increase in participant attendance and participation, likely influenced by the positive group dynamics. Moreover, participants organized potlucks within their groups at the conclusion of sessions. Such synergistic effects contribute to the overall productivity of the group, greater than the combined productivity of its individual members [78]. Thus, such sessions are likely to foster community and social support networks for pregnant women, an important aspect in promoting resilience. Social loafing where individuals exert less effort in a group setting compared to when working individually, is a drawback of group activities as it often results in unequal contributions among group members [79]. However, our interactive discussion and reflection-based approach encouraged every member to participate equally, thereby minimizing competition and promoting inclusivity. Moreover, the trainers were specifically trained to handle ground rules that were agreed upon by the group participants, no friction was observed between participants or between participants and trainers although we cannot exclude that some participants felt. Gençer et al. (2019) also showed that prior setting up of rules considering suggestions of the participants involved, allows a smooth run of the activity and has a positive impact on group dynamics [80]. Our participants were encouraged and facilitated to speak freely by persistent emphasis on respecting secrecy and confidentiality. Moreover, each session of SM-ART intervention consisted of engaging strategies that encouraged participants to reflect, recognize their own strengths and to develop coping mechanisms for overcoming obstacles during the thought-provoking sessions and later in their lives.

It is worth noting that all our participants obtained permission from their husbands and/or mothers-in-law prior to participating in the study. This highlights their limited autonomy and lack of empowerment. In our cultural context, mothers-in-law are often perceived as influential figures in their children's lives, particularly post-marriage. Numerous studies have indicated that the influence of mothers-in-law can significantly affect marital harmony within couples [81, 82]. Therefore, it is important to consider that women who were successful in persuading their husbands and families to participate may already possess a certain level of empowerment, while those who faced challenges in obtaining permission may be in greater need of such training.

This intervention was successfully facilitated by CMWs which is another strength in our study. It’s highly replicable, socio-culturally relevant, and transferable to the clinical environment. Midwifery-led education classes have been adopted widely as a method of supporting wellbeing, preventing onset of anxiety or depression and better child health outcomes [83]. Evans et al. [84] advocate providing midwives with suitable training to become competent and skilled at identifying mental health difficulties and delivering the right interventions to pregnant women [84]. In our study, the CMWs had a two-week training course to ensure that they could guide the sessions independently. They were also required to re-demonstrate the sessions and were evaluated by the research team as to their ability to meet the anticipated and unexpected challenges. This assured quality provision of the modules. Our intervention is also planned as a train-the-trainer method, which enables the quick expansion of knowledge and abilities and is also a cost-efficient way to offer training to a big group of people.

This study is the first of its kind to evaluate the effectiveness of an innovative, and culturally adapted intervention to reduce depressive symptoms and foster resilience among pregnant women. It was conducted during the COVID 19 pandemic which threatened to halt the study. Yet, to the credit of all concerned, every effort was made to overcome the challenges associated with implementing a group-based intervention in that challenging context. The strengths of the study included successful block randomization which was accurate at achieving balanced intervention and control groups at baseline and protecting the validity of the results. Moreover, blinding the data collector to the allocation of group was an important methodological feature to minimize researcher bias in collecting the data to ensure the internal validity of the current study.

Regarding limitations, firstly, the number of participants in each group is limited so future research should be conducted with larger samples to produce more reliable and generalizable results. Secondly, our outcome measures were self-reported which may have led to reporting bias; however, this should have been similar for both groups. Thirdly, the intervention sessions were led by two independent midwifery pairs so the individual personalities and characteristics might have influenced the method of delivery, the understanding or interpretation of the intervention. Fourthly, our follow-up assessment was conducted soon after the intervention which may have been insufficient to detect long-term gains so repeated measures testing after intervention and after delivery is a recommendation for future studies.

The findings of this study can also be used to replicate such studies in more settings. Moreover, the scalability of this intervention holds promise for widespread implementation, potentially it could be transformed into a standard practice during antenatal care. Scaling up this intervention has the potential to positively impact the mental well-being of women on a broader scale, extending its benefits to a larger demographic. This will build the latest, region specific, and relevant data. In addition, this may persuade policy makers to extend mental health support to pregnant women, which will increase service accessibility. While midwives undergo intensive training to deliver these interventions, the feasibility of this model lies in their dual role: they receive training themselves while also serving as educators. As primary caregivers in antenatal clinics, midwives are uniquely positioned to pass on knowledge and skills to their colleagues for conducting these sessions, as well as to expectant mothers during clinic visits. These intervention sessions can be integrated into routine antenatal visits, utilizing midwives to deliver them as a standard part of care during appointments. Our findings also suggest incorporating the idea of positive mental health with an emphasis on safe motherhood in the academic midwifery curriculum. The international trends are now focusing on positive mental health as opposed to mental health deficit, so this should also be reflected in midwifery curricula of Pakistan. Because midwifery curriculum of Pakistan is grossly limited to physical aspects of pregnancy and labor but reach of midwives is very high so empowering them and making them more equipped to mental health will bring change on larger scale. The lack of Pakistan-specific data has been significant barrier to advancing mental health care access in the country and implementing effective interventions. However, this study lays a solid foundation for future research initiatives, particularly in testing interventions like SM-ART. These interventions could be adapted for online or electronic media platforms, targeting pregnant women with better socioeconomic status who have access to electronic media. For future studies, assessing the effectiveness of this intervention by involving both parents could be done. Given that both partners (wife and husband) share a unique experience during the pregnancy period, it is crucial to understand how this intervention impacts not only individual outcomes but also the dynamics within the family unit.

## Conclusion

Globally, the growing magnitude of mental health issues demands development and implementation of sustainable interventions, in broader clinical settings. The findings of this study provide support for adopting the SM-ART intervention to promote resilience and reduce depressive symptoms among pregnant women. Hence, inclusion of such interventions in public health initiatives, particularly in countries with limited resources like Pakistan, may help to improve the mental health of women and foster the development of healthy families and societies. We recommend every woman who seeks antenatal care should be encouraged to participate in this intervention at least once during her pregnancy, whereby women will have the opportunity to share their feelings and concerns in a safe platform environment and receive relevant interventions to promote resilience and decrease depressive symptoms.

### Supplementary Information

Supplementary Material 1.

## Data Availability

The datasets used and/or analyzed during the current study are available from the corresponding author on reasonable request.
