# Supplementary material for: Promoting mental wellbeing in pregnant women living in Pakistan with the Safe Motherhood—Accessible Resilience Training (SM-ART) intervention: a randomized controlled trial
Source: BMC Pregnancy Childbirth. 2024 Jun 29;24:452. doi: 10.1186/s12884-024-06629-2 (PMC11218085; doi:10.1186/s12884-024-06629-2)
Supplement: Supplementary file 1 — Supplementary Material 1. [file 12884_2024_6629_MOESM1_ESM.docx]

| **Supplementary Table 1: Sociodemographic, pregnancy, and family characteristics of study participants** | | | | | | |
| --- | --- | --- | --- | --- | --- | --- |
|  | **Intervention** | **Control** | **p value** | **Retained** | **Dropouts** | **p value** |
|  | N=100 | N=100 |  | N=154 | N=46 |  |
| ***Demographics*** | | | | | | |
| Age |  |  | 0.32 |  |  | 0.039* |
| <20 years | 18 (18.00%) | 12 (12.00%) |  | 20(13%) | 10(21.7%) |  |
| 20-24 | 52 (52.00%) | 47 (47.00%) |  | 74(48.1%) | 25(54.3%) |  |
| 25-29 | 19 (19.00%) | 23 (23.00%) |  | 32(20.8%) | 10(21.7%) |  |
| >30 | 11 (11.00%) | 18 (18.00%) |  | 28(18.2%) | 1(2.2%) |  |
| Years of schooling |  |  | 0.42 |  |  | 0.787 |
| No formal education | 30 (30.00%) | 39 (39.00%) |  | 56(36.4%) | 13(28.3%) |  |
| Primary (1-5 yrs) | 27 (27.00%) | 24 (24.00%) |  | 38(24.7%) | 13(28.3%) |  |
| Secondary (6-10 yrs) | 32 (32.00%) | 31 (31.00%) |  | 47(30.5%) | 16(34.8%) |  |
| Post-Secondary (>10 yrs) | 11 (11.00%) | 6 (6.00%) |  | 13(8.4%) | 4(8.7%) |  |
| Mother Tongue (proxy for ethnicity) |  |  | 0.70 |  |  | 0.997 |
| Sindhi | 11 (11.00%) | 13 (13.00%) |  | 19(12.3%) | 5(10.9%) |  |
| Urdu | 26 (26.00%) | 19 (19.00%) |  | 34(22.1%) | 11(23.9%) |  |
| Punjabi | 11 (11.00%) | 10 (10.00%) |  | 16(10.4%) | 5(10.9%) |  |
| Pushto | 31 (31.00%) | 32 (32.00%) |  | 49(31.8%) | 14(30.4%) |  |
| Others | 21(21.00%) | 26 (26.00%) |  | 36(23.4%) | 11(23.9%) |  |
| Financially Empowered |  |  | 0.80 |  |  | 0.999 |
| Yes | 9 (9.00%) | 7 (7.00%) |  | 13(8.4%) | 3(6.5%) |  |
| No | 91 (91.00%) | 93 (93.00%) |  | 141(91.6%) | 43(93.5%) |  |
| ***Pregnancy Related Variables*** | | | | | | |
| Gestational age (in weeks) |  |  | 0.572 |  |  | 0.245 |
| Second Trimester | 51 (51.00%) | 47 (47.00%) |  | 79(51.3%) | 19(41.3%) |  |
| Third Trimester | 49 (49.00%) | 53 (53.00%) |  | 75(48.7%) | 27(58.7%) |  |
| First pregnancy (Primigravida) |  |  | 0.88 |  |  | 0.852 |
| Yes | 29 (29.00%) | 28 (28.00%) |  | 45(29.2%) | 12(26.1%) |  |
| No | 71 (71.00%) | 72 (72.00%) |  | 109(70.8%) | 34(73.9%) |  |
| History of miscarriage |  |  | 0.63 |  |  | 0.707 |
| Yes | 26 (26.00%) | 29 (29.00%) |  | 41(26.6%) | 14(30.4%) |  |
| No | 74 (74.00%) | 71 (71.00%) |  | 113(73.4%) | 32(69.6%) |  |
| History of stillbirth |  |  | 0.44 |  |  | 0.999 |
| Yes | 2 (2.00%) | 5 (5.00%) |  | 6(3.9%) | 1(2.2%) |  |
| No | 98 (98.00%) | 95 (95.00%) |  | 148(96.1%) | 45(97.8%) |  |
| Medical complications in current pregnancy (self-reported) |  |  | 0.67 |  |  | 0.999 |
| Yes | 51 (51.00%) | 48 (48.00%) |  | 76(49.5%) | 23(50%) |  |
| No | 49 (49.00%) | 52 (52.00%) |  | 78(50.5%) | 23(50%) |  |
| Intended or planned pregnancy |  |  | 0.74 |  |  | 0.18 |
| Yes | 74 (74.00%) | 76 (76.00%) |  | 119(77.3%) | 31(67.4%) |  |
| No | 26 (26.00%) | 24 (24.00%) |  | 35(22.7%) | 15(32.6%) |  |
| ***Marriage Related Variables*** | | | | | | |
| Participants type of marriage |  |  | 0.019* |  |  | 0.788 |
| Arranged | 84 (84.00%) | 95 (95.00%) |  | 137(89%) | 42(91.3% |  |
| Self-choice | 16 (16.00%) | 5 (5.00%) |  | 17(11%) | 4(8.7%) |  |
| Consanguineous marriage |  |  | 0.39 |  |  | 0.732 |
| Yes | 57 (57.00%) | 63 (63.00%) |  | 91(59.1%) | 29(63%) |  |
| No | 43 (43.00%) | 37 (37.00%) |  | 63(40.9%) | 17(37%) |  |
| Duration of marriage in years |  |  | 0.092 |  |  | 0.089 |
| 1-2 years | 42 (42.00%) | 31 (31.00%) |  | 54(35.1%) | 19(41.3%) |  |
| 3-5 years | 28 (28.00%) | 26 (26.00%) |  | 37(24.0%) | 17(37.0%) |  |
| 6-10 years | 24 (24.00%) | 27 (27.00%) |  | 43(27.9%) | 8(17.4%) |  |
| more than 10 years | 6 (6.00%) | 16 (16.00%) |  | 20(13.0%) | 2(4.3%) |  |
| ***Family Related Variables*** | | | | | | |
| Participants spouse employed |  |  | 0.59 |  |  | 0.752 |
| Yes | 91 (91.00%) | 94 (94.00%) |  | 143(92.9%) | 42(91.3%) |  |
| No | 9 (9.00%) | 6 (6.00%) |  | 11(7.1%) | 4(8.7%) |  |
| Participants family type |  |  | 0.091 |  |  | 0.719 |
| Joint | 75 (75.00%) | 64 (64.00%) |  | 108(70.1%) | 31(67.4%) |  |
| Nuclear | 25 (25.00%) | 36 (36.00%) |  | 46(29.9%) | 15(32.6%) |  |
| Number of members in a household |  |  | 0.84 |  |  | 0.009 |
| Two people | 10 (10.00%) | 7 (7.00%) |  | 11(7.1%) | 6(13.0%) |  |
| Three to five people | 27 (27.00%) | 31 (31.00%) |  | 39(25.3%) | 19(41.3%) |  |
| Six to nine people | 30 (30.00%) | 29 (29.00%) |  | 54(35.1%) | 5(10.9%) |  |
| More than ten people | 33 (33.00%) | 33 (33.00%) |  | 50(32.5%) | 16(34.8%) |  |
| Number of Alive Children |  |  | 0.56 |  |  | 0.253 |
| 1-2 children | 45 (45.00%) | 39 (39.00%) |  | 60(39%) | 24(52.2%) |  |
| 3-5 children | 12 (12.00%) | 17 (17.00%) |  | 25(16.2%) | 4(8.7%) |  |
| >5 children | 4 (4.00%) | 7 (7.00%) |  | 10(6.5%) | 1(2.2) |  |
| Not applicable | 39 (39.00%) | 37 (37.00%) |  | 59(38.3%) | 17(37.0%) |  |
| Household monthly income (Median and IQR) | 17,500 (14,000) | 20,000 (11,0000) | 0.60 | 18000(12250) | 20000(13000) | 0.768 |
| Own house |  |  | 0.67 |  |  | 0.242 |
| Yes | 48 (48.00%) | 45 (45.00%) |  | 68(44.2%) | 25(54.3%) |  |
| No | 52 (52.00%) | 55 (55.00%) |  | 86(55.8%) | 21(45.7%) |  |
| Own vehicle for transportation |  |  | 0.66 |  |  | 0.223 |
| Yes | 35 (35.00%) | 38 (38.00%) |  | 60(39.0%) | 13(28.3%) |  |
| No | 65 (65.00%) | 62 (62.00%) |  | 94(61.0%) | 33(71.7%) |  |
| ***Social Life Variables*** | | | | | |  |
| Number of friends |  |  | 0.13 |  |  | 0.649 |
| Zero | 65 (65.00%) | 75 (75.00%) |  | 107(69.5%) | 33(71.7%) |  |
| 1-2 | 23 (23.00%) | 12 (12.00%) |  | 26(16.9%) | 9(19.6%) |  |
| 3 or more | 12 (12.00%) | 13 (13.00%) |  | 21(13.6%) | 4(8.7%) |  |
| Participate in social or voluntary activities or services |  |  | 0.54 |  |  | 0.834 |
| No | 96 (96.00%) | 93 (93.00%) |  | 26(16.9%) | 9(19.6%) |  |
| Yes | 4 (4.00%) | 7 (7.00%) |  | 128(83.1%) | 37(80.4%) |  |
| ***Emotional Stress*** | | | | | |  |
| Ability to manage financial demands |  |  | 0.67 |  |  | 0.16 |
| Yes | 39 (39.00%) | 42 (42.00%) |  | 55(35.7%) | 26(56.5%) |  |
| No | 61 (61.00%) | 58 (58.00%) |  | 99(64.3%) | 20(43.5%) |  |
| Feeling strain in marital life |  |  | 0.77 |  |  | 0.664 |
| Yes | 7 (7.00%) | 5 (5.00%) |  | 95(61.7%) | 30(65.2%) |  |
| No | 93 (93.00%) | 95 (95.00%) |  | 59(38.3%) | 16(34.8%) |  |
| Feeling strain in social life or with family member |  |  | 0.28 |  |  | 0.999 |
| Yes | 5 (5.00%) | 10 (10.00%) |  | 9(5.8%) | 2(4.3%) |  |
| No | 95 (95.00%) | 90 (90.00%) |  | 145(94.2%) | 44(95.7%) |  |
| ***Outcomes*** | | | | | | |
| Resilience (14-98) | 75.25 (13.54) | 74.23 (14.34) | 0.606 | 76.08(13.69) | 70.246(13.89) | 0.015* |
| Marital Adjustment (2-158) | 121.00 (29.18) | 125.67 (22.02) | 0.203 | 123.13(25.44) | 124.02(27.61) | 0.838 |
| Pregnancy related anxiety (0-30) | 15.23 (6.33) | 15.85 (6.42) | 0.493 | 15.59(6.41) | 15.37(6.28) | 0.835 |
| Depression (0-30) | 11.48 (7.53) | 11.99 (7.38) | 0.629 | 11.81(7.47) | 11.5(7.40) | 0.807 |

|  |  |  |  |  |  |  |
| --- | --- | --- | --- | --- | --- | --- |
| **Supplementary Table 2: Comparison of post intervention scores of primary and secondary outcome between intervention and control group** | | | | | | |
| ***Outcomes*** | ***Complete Data (n=200)*** | | | ***Participants excluding dropouts(n=154)*** | | |
|  | **Intervention** | **Control** | ***p value***^Ϯ^ | **Intervention** | **Control** | ***p value***^Ϯ^ |
|  | N=100 | N=100 |  | N=77 | N=77 |  |
|  | Mean (SD) | Mean (SD) |  | Mean (SD) | Mean (SD) |  |
| Resilience (14-98) | 82.68 (14.54) | 75.77 (13.96) | 0.001* | 87.12 (11.21) | 76.69 (14.24) | <0.001* |
| Marital Adjustment (2-158) | 123.02 (28.47) | 123.57 (24.76) | 0.884 | 125.04 (26.07) | 121.12 (26.75) | 0.358 |
| Pregnancy related anxiety (0-30) | 13.79 (6.04) | 14.39 (6.32) | 0.494 | 13.19 (5.95) | 14.22 (6.27) | 0.299 |
| Depression (0-30) | 8.86(6.67) | 10.98 (7.56) | 0.037* | 7.97 (6.28) | 11.29 (7.88) | 0.005* |
| *Note: Ϯ Independent t-test.*    Ϯ Independent t-test. | | | | | | |
|  |  |  |  |  |  |  |

| **Supplementary Table 3: Comparison of primary and secondary outcome scores within intervention group after 6 weeks** | | | | | | |
| --- | --- | --- | --- | --- | --- | --- |
|  | ***Complete Data (n=200)*** | | | ***Participants excluding dropouts(n=154)*** | | |
|  | **Pre** | **Post** | ***p value***^Ϯ^ | **Pre** | **Post** | ***p value***^Ϯ^ |
|  | N=100 | N=100 |  | N=77 | N=77 |  |
| ***Outcomes*** | Mean (SD) | Mean (SD) |  | Mean (SD) | Mean (SD) |  |
| Resilience (14-98) | 75.25(13.54) | 82.68 (14.54) | <0.001* | 77.47 (12.41) | 87.12 (11.21) | <0.001* |
| Marital Adjustment (2-158) | 121.0 (29.18) | 123.02(28.47) | 0.284 | 122.42 (27.23) | 125.04 (26.07) | 0.285 |
| Pregnancy related Anxiety (0-30) | 15.23 (6.33) | 13.79 (6.04) | 0.01* | 15.06 (6.43) | 13.19 (5.95) | 0.01* |
| Depression (0-30) | 11.48 (7.53) | 8.86 (6.67) | <0.001* | 11.01 (7.34) | 7.97 (6.28) | <0.001* |
| *Note: Ϯ Paired t-test.* | | | | | | |
|  | | | | | | |
| **Supplementary Table 4: Comparison of primary and secondary outcome scores within control group after 6 weeks** | | | | | | |
|  | ***Complete Data (n=200)*** | | | ***Participants excluding dropouts(n=154)*** | | |
|  | **Pre** | **Post** | ***p value***^Ϯ^ | **Pre** | **Post** | ***p value***^Ϯ^ |
|  | N=100 | N=100 |  | N=77 | N=77 |  |
| ***Outcomes*** | Mean (SD) | Mean (SD) |  | Mean (SD) | Mean (SD) |  |
| Resilience (14-98) | 74.23 (14.34) | 75.77 (13.96) | 0.215 | 74.69 (14.81) | 76.69 (14.24) | 0.215 |
| Marital Adjustment (2-158) | 125.67 (22.02) | 123.57 (24.76) | 0.308 | 123.84 (26.67) | 121.12 (26.75) | 0.309 |
| Pregnancy related Anxiety (0-30) | 15.85 (6.42) | 14.39 (6.32) | 0.010* | 16.12 (6.39) | 14.22 (6.27) | 0.009* |
| Depression (0-30) | 11.99 (7.38) | 10.98 (7.56) | 0.075 | 12.60(7.56) | 11.29 (7.88) | 0.074 |
| *Note: Ϯ Paired T test* | | | | | | |

| **Supplementary Table 5a: Variables significantly predicting Resilience Score difference** | | | | | | |
| --- | --- | --- | --- | --- | --- | --- |
|  | ***Complete Data (n=200)*** | | | ***Participants excluding dropouts(n=154)*** | | |
| ***Variables*** | ***Beta Coeff*** | ***SE*** | ***p value*** | ***Beta Coeff*** | ***SE*** | ***p value*** |
| Intercept | 29.81 | 4.42 | 0.00 | 45.115 | 5.2034 | 0.000 |
| Assigned Group |  |  |  |  |  |  |
| Intervention | 6.28 | 1.60 | 0.00 | 9.254 | 1.8418 | <0.001 |
| Control (Ref) | 0.00 | . |  | 0^a^ |  |  |
| Resilience Score at Baseline | -0.38 | 0.06 | 0.00 | -0.577 | 0.0675 | 0.000 |

| **Supplementary Table 5b: Variables significantly predicting Depression Score Difference** | | | | | | |
| --- | --- | --- | --- | --- | --- | --- |
|  | ***Complete Data (n=200)*** | | | ***Participants without dropouts(n=154)*** | | |
| ***Variables*** | ***Beta Coeff*** | ***SE*** | ***p value*** | ***Beta Coeff*** | ***SE*** | ***p value*** |
| Intercept | 9.590 | 2.322 | 0.000 | 11.955 | 3.087 | 0.000 |
| Assigned Group |  |  |  |  |  |  |
| Intervention | -1.730 | 0.725 | 0.017 | -2.259 | 0.914 | 0.013 |
| Control (Ref) | 0.000 | . |  | 0^a^ |  |  |
| Resilience Score at Baseline | -0.079 | 0.027 | 0.004 | -0.093 | 0.035 | 0.008 |
| Depression Score at Baseline | -0.394 | 0.051 | 0.000 | -0.499 | 0.065 | 0.000 |
|  |  |  |  |  |  |  |
|  |  |  |  |  |  |  |
| **Supplementary Table 5c: Variables significantly predicting Marital Adjustment Score Difference** | | | | | | |
|  | ***Complete Data (n=200)*** | | | ***Participants excluding dropouts(n=154)*** | | |
| ***Variables*** | ***Beta Coeff*** | ***SE*** | ***p value*** | ***Beta Coeff*** | ***SE*** | ***p value*** |
| Intercept | 21.79 | 8.07 | 0.007 | 22.27 | 10.63 | 0.036 |
| Assigned Group |  |  |  |  |  |  |
| Intervention | 2.68 | 2.48 | 0.279 | 3.85 | 3.24 | 0.234 |
| Control (Ref) | 0.00 | . | . | 0^a^ |  |  |
| Resilience Score at Baseline | 0.30 | 0.10 | 0.001 | 0.33 | 0.12 | 0.007 |
| Marital Adjustment Score at baseline | -0.33 | 0.05 | 0.000 | -0.40 | 0.07 | 0.000 |
| Working Status |  |  |  | - | - | - |
| Yes | -10.65 | 4.68 | 0.023 | - | - | - |
| No (Ref) | 0.00 | . | . | - | - | - |
| Problems in current pregnancy |  |  |  | - | - | - |
| Yes | -7.33 | 2.48 | 0.033 | - | - | - |
| No (Ref) | 0.00 | . | . | - | - | - |

|  |  |  |  |  |  |  |
| --- | --- | --- | --- | --- | --- | --- |
| **Supplementary Table 5d: Variables significantly predicting Anxiety Score Difference** | | | | | | |
|  | ***Complete Data (n=200)*** | | | ***Participants excluding dropouts(n=154)*** | | |
| ***Variables*** | ***Beta Coeff*** | ***SE*** | ***p value*** | ***Beta Coeff*** | ***SE*** | ***p value*** |
| Intercept | 4.85 | 1.11 | 0.00 | 5.823 | 1.2112 | 0.000 |
| Assigned Group |  |  |  |  |  |  |
| Intervention | -0.30 | 0.65 | 0.64 | -0.341 | 0.8278 | 0.681 |
| Control (Ref) | 0 | . |  | 0^a^ |  |  |
| Anxiety Score at baseline | -0.47 | 0.06 | 0.00 | -0.620 | 0.0743 | 0.000 |
| Depression Score at baseline | 0.12 | 0.05 | 0.02 | 0.180 | 0.0639 | 0.005 |
| Problems in current pregnancy |  |  | 0.00 | - | - | - |
| Yes | 1.93 | 0.66 |  | - | - | - |
| No | 0 |  |  | - | - | - |
| Pregnancy Decision |  |  | 0.03 | - | - | - |
| Own | -1.62 | 0.76 |  | - | - | - |
| Husband | 0 |  |  | - | - | - |
|  |  |  |  |  |  |  |
|  |  |  |  |  |  |  |
